# Evaluation of Multimode Color Doppler Flow Imaging in the Diagnosis of Solid Renal Tumor

**DOI:** 10.1155/2021/6656877

**Published:** 2021-04-01

**Authors:** Ming Chen, Xiaohong Fu, Yan Shen

**Affiliations:** Department of Medical Ultrasound, Pudong Gongli Hospital, Shanghai 200135, China

## Abstract

**Background:**

Renal cell carcinoma is one of the most common malignant tumors in urinary system, seriously affecting people's health and life. This study aimed to evaluate the clinical value of multi-mode color Doppler flow imaging for diagnosis of solid renal tumor.

**Methods:**

Sixty-six renal solid tumors from 63 patients were examined by color Doppler flow imaging (CDFI), power Doppler flow imaging (PDFI), superb microvascular imaging (SMI), and contrast-enhanced ultrasound (CEUS) before surgery. The diagnostic efficacy of the four methods was compared by determining blood flow grade and ring-like blood flow with Adler's method. Chi-square test and Fisher's test were performed to compare the results of sensitivity and specificity among four methods.

**Results:**

Statistically significant differences in blood flow grade and ring-like blood flow were observed between benign and malignant renal tumors as detected by SMI and CEUS (*P* < 0.05), whereas no difference was found as detected by CDFI and PDFI (*P* > 0.05). The results indicated that the sensitivity and specificity of SMI (82.46%, 88.89%) and CEUS (84.21%, 88.89%) were higher than those of CDFI (42.11%, 66.67%) and PDFI (47.37%, 77.78%). Compared with the abilities of CDFI and PDFI, SMI and CEUS can better display the micro-blood flow in the tumors and evaluate the blood flow grading, which indicated that SMI and CEUS may have high values in the differential diagnosis of benign and malignant solid renal tumors.

**Conclusion:**

SMI and CEUS can improve the sensitivity and specificity of the diagnosis of benign and malignant renal tumors and have a high application value.

## 1. Background

Renal cell carcinoma (RCC) is the third most common cancer of the urinary system after prostate and bladder [1]. Since the patients usually have no obvious symptoms, the renal malignant tumors are detected late and the prognosis is poor [2, 3]. Ultrasound has been widely used in the detection of renal tumors since it is inexpensive, noninvasive, and repeatable [4]. However, the sonographic features of benign and malignant renal tumors are similar, and it is easy to miss or misdiagnose the disease if only examined by two-dimensional ultrasound [5].

Malignant tumors usually maintain their growth through continuous neovascularization, which provides a pathological indication for differentiating the benign and malignant tumors. Whether ultrasound can accurately detect neovascularization in mass is of great significance in differential diagnosis [6]. Power Doppler flow imaging (PDFI) is a technique used to detect the blood flow information, specifically the moving red blood cells, which is not influenced by the direction of blood flow [7]. Besides, PDFI is more sensitive than CDI but is incapable of determining velocity and direction of flow. Contrast-enhanced ultrasound (CEUS) is a technique using microbubbles, which are less than 10 *μ*m in diameter and can pass through capillaries freely, to display microcirculation perfusion [8]. It is currently the “golden standard” for examination of microvasculature with ultrasound. Superb microvascular imaging (SMI) is a new Doppler technique and is informative for evaluating very slow blood flow state [9]. Adaptive algorithm allows for imaging microvessels with low velocity in the absence of a contrast agent. SMI has been reported to be accurate and efficient in diagnosing breast tumors and liver lesions [10, 11].

In the current study, color Doppler flow imaging (CDFI), PDFI, CEUS, and SMI were used to detect the blood vessels in solid renal tumors, and the results of blood flow grading and ring-like blood flow signal were analyzed and compared to explore the diagnostic value of these four ultrasound blood flow imaging techniques for solid renal tumors.

## 2. Materials and Methods

### 2.1. Inclusion of Patients

**Figure 1:**
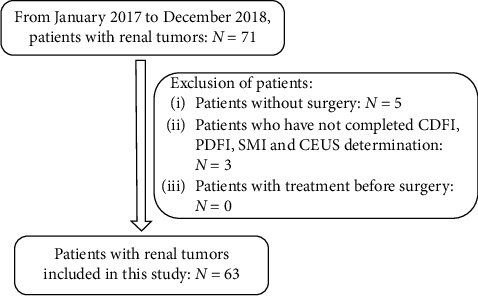
The inclusion and exclusion criteria of patients.

Patients with renal tumor in our hospital from January 2017 to December 2018 were included. The study protocol was approved by the Ethics Committee of Pudong Gongli Hospital, and informed consents were obtained from all the cases included in this study. The inclusion and exclusion criteria of patients are shown in Figure 1. Before the surgery, CDFI, PDFI, SMI, and CEUS were performed and all the tumors underwent pathological examination after surgical excision.

It was found that a total of sixty-six solid renal masses from 63 patients (37 males and 26 females) were included. The age ranged from 22 to 85 years and the average age was 59.03 ± 14.05 years. There were 27 tumors on the left side and 39 tumors on the right side. According to statistical analysis, the average diameter of malignant tumors was 4.29 ± 3.08 cm and the average diameter of benign tumors was 8.51 ± 3.80 cm.

### 2.2. Procedures

Ultrasound instruments used were Toshiba Aplio 500, Philips IU Elite color Doppler ultrasound instrument (frequency 1.0–6.0 MHz), with convex array probe. The images with displayed through PDFI, SMI imaging software, and contrast software.

The patient exposed the abdomen with a supine or lateral position. Firstly, conventional two-dimensional ultrasound was performed to observe the location, size, and shape of the tumors from different angles and the maximum diameter of the tumors was measured. Then CDFI, PDFI, and monochrome SMI were conducted to observe the blood flow in the tumors. The gray-scale gain was 75–90 dB. The measuring scales of CDFI and SMI were 9.8–19.6 cm/s and 1.5–2.4 cm/s, respectively.

A section which clearly showed both tumors and the surrounding kidney tissue was chosen for contrast-enhanced ultrasound (CEUS). Sono Vue solution (1.2 mL, dissolved by 5 mL saline) was used as ultrasound contrast agent. The whole procedure continued for more than 2 minutes, and the relative images were recorded. The imaging parameters of CEUS were mechanical index (MI) < 0.05, and dynamic range 40–50 dB.

### 2.3. Imaging Analysis

Blood flow analysis of tumors included the blood flow grading and the presence of peripheral ring-like blood flow. According to Adler's method [12], the blood flow signals were graded as follows: grade 0 was no blood flow signals in tumors; grade I was 1-2 dot-like or fine rod-like blood flow signals in tumors; grade II was 3-4 dot-like or one important blood vessel, whose length was close to or exceeded the radius of tumors; grade III was more than 5 dot-like or 2 longer blood vessels. Peripheral ring-like blood flow: ring-like or approximate ring-like blood flow signals appear around the tumor. All cases were diagnosed by two senior doctors with more than three years of contrast-enhanced ultrasound experience.

### 2.4. Statistical Analysis

SPSS17.0 software (SPSS Inc., Chicago, IL, USA) was used for statistical analysis. Data were presented as number or percentage. Pearson's *χ*^2^ test and two-tailed Fisher's exact test were used for the comparison between benign and malignant groups in each examination method. The ROC curve was used to compare the efficiency of these four methods in differentiating benign and malignant renal, and the differences between groups were compared by *Z* test method. *P* < 0.05 was considered as statistical significance.

## 3. Results

### 3.1. Pathological Findings

Among 57 malignant tumors, 43 were clear cell carcinoma, 8 were papillary cell carcinoma, 4 were chromophobe cell carcinoma, and 2 were adult nephroblastoma. Among 9 benign tumors, 7 were angiomyolipoma and 2 were inflammatory granuloma. Of the 43 clear cell carcinoma, 8 were Furhman I, 29 were Furhman II, 5 were Furhman III, and 1 was Furhman IV.

### 3.2. Comparison of Four Techniques in Detecting Blood Flow Grading in Benign and Malignant Renal Tumors

To evaluate the effects of four techniques, the blood flow grade between benign and malignant was compared by Adler's method. There was no significant difference in blood flow grade between benign and malignant tumors examined by CDFI and PDFI (*P* = 0.109, *P* = 0.304), whereas the difference in blood flow grade between benign and malignant tumors detected using SMI and CEUS (*P* = 0.009, *P* = 0.011) was significant (Table 1). The results indicated that SMI and CEUS could better distinguish the blood flow grade between benign and malignant tumors compared with CDFI and PDFI.

### 3.3. Comparison of Four Techniques in Detecting Ring-Like Blood Flow of Benign and Malignant Renal Tumors

**Figure 2:**
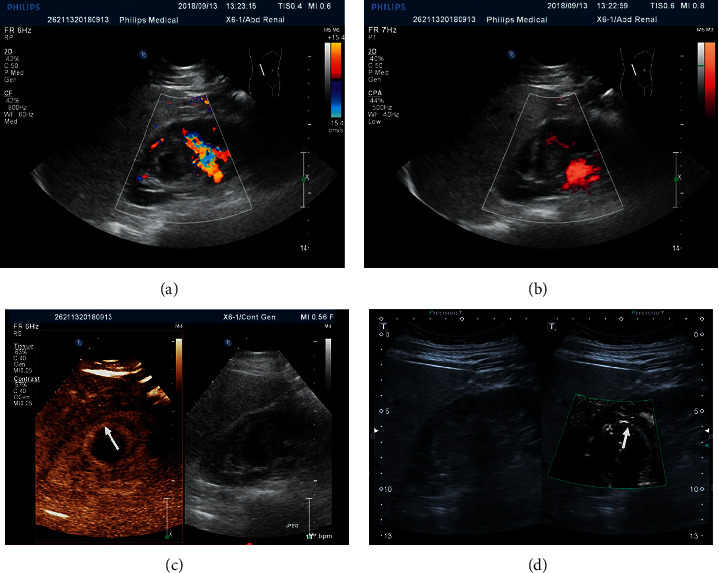
The ring-like blood flow signal images of clear cell renal carcinoma in the same patient detected by (a) CDFI, (b) PDFI, (c) CEUS, and (d) SMI.

Either CDFI or PDFI failed to detect ring-like blood flow signals around 9 benign tumors, while CEUS and SMI detected ring-like blood flow signals around 1 benign tumor. Among the total 57 malignant tumors with ring-like blood flow signals, CDFI detected 9 cases, PDFI detected 12 cases, CEUS detected 40 cases, and SMI detected 37 cases. In the same patient, CDFI and PDFI detected the malignant tumors without ring-like blood flow signals (Figures 2(a) and 2(b)), while CEUS and SMI detected significant ring-like blood flow signals (Figures 2(c) and 2(d)). These results indicated that, compared with CDFI and PDFI, CEUS and SMI had better effects to diagnose the malignant tumors in the same patient.

In addition, there was no significant difference in ring-like blood flow between the benign and malignant renal tumors as detected by CDFI and PDFI (*χ*^2^ = 1.521, *P* = 0.217; *χ*^2^ = 2.122, *P* = 0.145), while the level between benign and malignant renal tumors measured using SMI and CEUS (*χ*^2^ = 9.823, *P* = 0.003; *χ*^2^ = 9.157, *P* = 0.001) was evidently different (Table 2). These results demonstrated that SMI and CEUS could distinguish between benign and malignant renal tumors well.

### 3.4. Comparison of Diagnostic Efficacy of Four Techniques in Benign and Malignant Renal Tumors

**Figure 3:**
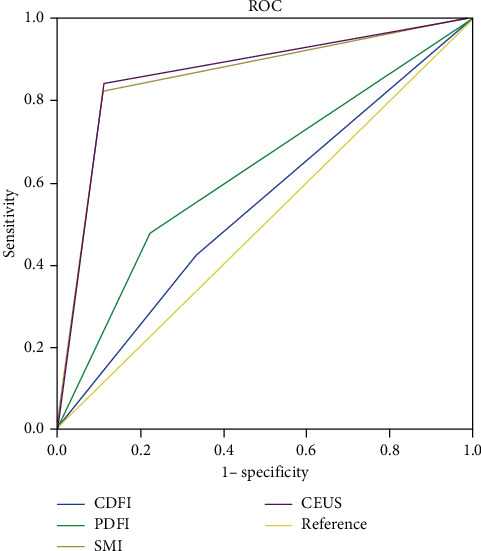
Receiver operating characteristic (ROC) curves for diagnosis of benign and malignant renal lesions by CDFI, PDFI, SMI, and CEUS. *X*-axis represents the specificity. *Y*-axis represents the sensitivity.

The sensitivity, specificity, and area under ROC curve of CDFI, PDFI, SMI, and CEUS in the diagnosis of benign and malignant renal tumors are shown in Table 3 and Figure 3. It was shown that SMI and CEUS are superior to CDFI and PDFI in the diagnosis of benign and malignant tumors, with statistical significance (*P* (CDFI vs. SMI) = 0.0001; *P* (CDFI vs. CEUS) = 0.0001; *P* (PDFI vs. SMI) = 0.0003; *P* (PDFI vs. CEUS) = 0.0002), whereas no significant differences were observed between SMI and CEUS (*P* = 0.3173), as well as between CDFI and PDFI (*P* = 0.1547).

## 4. Discussion

RCC, with steadily increasing incidence, is one of the most common malignant tumors in urinary system, seriously affecting people's health and life [13, 14]. The two-dimensional ultrasonographic features of benign and malignant tumors overlap partially, which reduce the diagnosis accuracy [15, 16]. It is well known that renal cell carcinoma is a tumor with abundant blood supply. Pathological studies showed that neovascularization with high density and disordered arrangement was found in the cancer tissue, and the number of blood vessels and blood flow grading are significantly higher than those of benign tumors [17, 18]. Previous studies have shown that microvessel density is related to the grade, metastasis, and prognosis of tumors [19, 20]. Therefore, it is of great significance to explore better methods to evaluate the microvascular system in renal parenchymal tumors for the early diagnosis and differential diagnosis of RCC.

In this study, Adler method was used to classify the blood flow of solid tumors. There was no significant difference between the blood flow classification of benign and malignant tumors as detected by CDFI and PDFI, whereas there was significant difference as detected by SMI and CEUS. CDFI is based on the relative motion between red blood cells and probes, which is described by computer pseudocolor technology. The blood flow signals are closely related to the direction and velocity of blood flow. Therefore, it is hard to distinguish the difference between low-speed blood flow and motion artifacts by CDFI [21]. Additionally, diagnosis of blood flow signals greatly depends on subjective judgements, so all cases were diagnosed by two senior doctors with more than three years of contrast-enhanced ultrasound experience in this study. It was found that only the blood flow signals with diameter >0.2 mm and high flow velocity can be detected. PDFI detects the blood flow information through detecting the echogenicity of blood, which is not affected by the direction of blood flow, while the ability to detect small and low-speed blood flow signals is poor [22]. Taken together, both CDFI and PDFI are insensitive to small neovascularization in malignant tumors. The blood flow classification of malignant tumors is mainly grade 0 and grade I.

SMI is a newly developed high-resolution blood flow imaging technique, which distinguishes tissue motion noise from real blood flow information, detects low-speed blood flow signals through signal processing technology, and clearly presents low-speed blood flow signals in microvessels [23, 24]. The images of SMI are at high frame rate, high spatial resolution, and less few motion artifacts and can display low-speed microvessels with diameter >0.1 mm. The microbubbles of contrast agent can reach the capillary bed of the lesion site along with the blood circulation and produce a large amount of strong echo scattering when contacted with red blood cells, thus increasing the acoustic impedance difference between blood vessels and tissues, improving the interface reflectance, sensitively displaying the microvessels of tumors, and improving the display of low-speed blood flow [25, 26]. Therefore, the blood classifications of malignant tumors detected by SMI and CEUS are mainly grades II and III. Compared with CDFI and PDFI, SMI, and CEUS are more sensitive and accurate in detecting blood signals of malignant tumors, which enhances the diagnostic accuracy of renal solid tumors.

Clear cell renal cell carcinoma is the most common malignant solid tumor in the kidney [27]. Previous studies have shown that ring-like blood flow signals can appear around malignant tumors, which is the specific manifestation of malignant tumors [28, 29]. The mechanism is that the pseudocapsule enclosing the tumors between malignant renal tumors and surrounding normal renal tissues is composed of a large number of fibrous tissues and capillaries [30]. In the current study, four techniques were used to detect the circumferential blood flow signals around the tumors. Among 57 malignant tumors, 37 were detected by SMI, 40 by CEUS, and only 9 by CDFI and 12 by PDFI, respectively. The detection rates of ring-like blood flow of SMI and CEUS were significantly higher than CDFI and PDFI; the difference was statistically significant (*P* < 0.05). Interestingly, both SMI and CEUS detected a ring-like blood signal in the same case of benign angiomyolipoma. Since the diameter of the tumor was 21 cm, it was considered that the phenomenon was caused by the overgrowth of the tumor and the compression of renal vessels, not by the capillaries in the pseudocapsule.

CEUS is currently recognized as the “gold standard” for the detection of microvessels by ultrasound, while it is only suitable for a single target [31]. If there are multiple tumors in one kidney or bilateral kidney tumors, repeat examinations or CEUS combined with CT/MRI are necessary, which may waste a lot of time and expenses. However, SMI is not affected by these objective factors. It is non-invasive, has no side effects, is relatively more economical, and can be implemented quickly and repeatedly [32]. In addition, our study showed that SMI has good consistency with CEUS in microvascular detection and diagnostic efficiency. This technique has a high translation value in clinical practice.

In addition, we diagnosed 57 malignant tumors in our study, including 43 clear cell carcinoma, 8 papillary cell carcinoma, 4 chromophobe cell carcinoma, and 2 adult nephroblastoma. In the diagnosed clear cell carcinoma, 41 of 43 cases had SMI blood flow signal grade 2-3, and only 2 cases had no blood flow signal due to the small tumor diameter (<1 cm). CEUS also failed to detect these two cases, and the reason might be that the tumor diameter was too small, leading to the unclear boundary between the contrast agent and the surrounding normal tissue. Furthermore, the microvessel density and vessel diameter of renal clear cell carcinoma were higher than those of normal renal, and both SMI and CEUS showed rich blood supply type; therefore, the diagnostic accuracy of SMI and CEUS was higher. However, for papillary cell carcinoma, chromophobe cell carcinoma, and adult nephroblastoma, because the blood supply in the tumor is insufficient, they cannot be distinguished by vascular density alone. However, due to the compression of normal surrounding tissues by tumor expansion and growth, it is easy to induce false capsule. Among the four different detection methods, SMI and CEUS are also more sensitive to the detection of false capsule. Taken together, SMI and CEUS may be better for the diagnosis of solid renal tumor.

However, there are some limitations in this study. The different sizes and pathologies of tumors were not classified. Besides, the number of samples is small and further studies with larger samples need to verify the conclusion. In addition, renal oncocytoma is a common benign nephrocytoma and may have potential adverse effects on patients; therefore, further studies should be further explored on the diagnosis of renal oncocytoma.

## 5. Conclusions

In conclusion, compared with PDFI and CDFI, SMI, and CEUS can more efficiently and accurately classify the blood flow and detect the peripheral ring-like blood flow signals. SMI and CEUS can improve the sensitivity and specificity of the diagnosis of benign and malignant renal tumors and have a high application value.

## Figures and Tables

**Table 1:** Blood flow grade in 66 benign and malignant renal lesions by CDFI, PDFI, SMI, and CEUS.

Examination method	The grade of Adler (*n*)				*X* ^2^	*P*
	Grade 0	Grade 1	Grade 2	Grade 3		
CDFI					6.057	0.109
Benign	4	4	1	0		
Malignant	8	25	17	7		
PDFI					3.634	0.304
Benign	3	3	3	0		
Malignant	7	23	19	8		
SMI					11.575	0.009
Benign	2	4	3	0		
Malignant	2	8	32	15		
CEUS					11.162	0.011
Benign	2	3	4	0		
Malignant	1	10	27	19		

*P* was calculated by Pearson's *χ*^2^ test. *P* < 0.05 was considered as statistical significance.

**Table 2:** Ring-like blood flow in 66 benign and malignant renal tumors by color Doppler flow imaging (CDFI), power Doppler flow imaging (PDFI), superb microvascular imaging (SMI), and contrast-enhanced ultrasound (CEUS).

Examination method	Ring-like blood flow		*X* ^2^	*P*
	+	−		
CDFI			1.521	0.217
Benign	0	9		
Malignant	9	48		
PDFI			2.122	0.145
Benign	0	9		
Malignant	12	45		
SMI			9.823	0.003
Benign	1	8		
Malignant	37	20		
CEUS			9.157	0.001
Benign	1	8		
Malignant	40	17		

*P* was calculated by Pearson's *χ*^2^ test. *P* < 0.05 was considered as statistical significance.

**Table 3:** The diagnostic value of CDFI, PDFI, SMI, and CEUS in benign and malignant renal lesions.

Methods	Accuracy (%)	Sensitivity (%)	Specificity (%)	Positive predictive value (%)	Negative predictive value (%)	AUC (95% CI)
CDFI	45.45	42.11	66.67	88.89	15.38	0.544 (0.343–0.774)
PDFI	51.52	47.37	77.78	93.10	18.92	0.626 (0.440–0.812)
SMI	83.88	82.46	88.89	97.92	44.44	0.857 (0.723–0.993)
CEUS	84.85	84.21	88.89	97.96	47.06	0.865 (0.734–0.997)

## Data Availability

The datasets used and/or analyzed during the current study are available from the corresponding author on reasonable request.

## Abbreviations

CDFI: Color Doppler flow imaging
PDFI: Power Doppler flow imaging
SMI: Superb microvascular imaging
CEUS: Contrast-enhanced ultrasound
RCC: Renal cell carcinoma.
